# Exploration of factors of digital photo hoarding behavior among university students and the mediating role of emotional attachment and fear of missing out

**DOI:** 10.3389/fpsyg.2025.1607274

**Published:** 2025-09-15

**Authors:** Wenjing Yu, Xueqin Chang

**Affiliations:** Department of Electronic and Information Engineering, Bozhou University, Bozhou, Anhui, China

**Keywords:** digital photo hoarding, fear of missing out, emotional attachment, SOR, PLS-SEM

## Abstract

With the widespread use of digital technology and devices, college students are prone to hoarding digital photos. Based on the SOR model, this study conducted a survey of 294 college students and used partial least squares structural equation modeling (PLS-SEM) to study the factors of digital photo hoarding among college students, as well as the mediating effects of emotional attachment and fear of missing on the relationship between various factors and digital photo hoarding behavior. The results revealed that emotional attachment, fear of missing out, interpersonal influence, life demand, and technological progress are important influencing factors for college students' digital photo hoarding behavior. In addition, Emotional attachment mediates the relationship between emotional needs, interpersonal influence, and technological progress with digital photo hoarding behaviors. Fear of missing out mediates the association between emotional needs, interpersonal influence, and technological progress, and digital photo hoarding behavior. Finally, we discuss the implication, limitations, and directions for future research and conclusion of this work.

## Introduction

According to Photutorial statistics, by 2024, it is expected that 1.94 trillion photos will be taken globally, with 5.3 billion photos taken every day, or 61,400 photos per second. There are approximately 14.3 trillion existing photos, and photos taken by smartphones account for 94% of all photos. Google Image Search can search about 136 billion pictures, 14 billion pictures are shared every day on social media, and Americans take 20 pictures every day on average (Agarwal et al., 2024). With the reduction of digital storage costs and the continuous expansion of storage capacity, as well as the enhancement of digital shooting and editing tools, people are hoarding more and more photos on devices such as mobile phones, hard drives, and cloud drives, and are unwilling to organize or delete them. A study shows that a 47 year old man takes about 1,000 photos every day and saves them all. Although he never looks at or uses these photos, he believes they will be useful in the future. Organizing these photos left the man very frustrated and time-consuming, taking 3–5 h a day, seriously affecting his normal life (Bozaci and Gökdeniz, 2020). College students are active users of social media and an important group for hoarding digital photos. The study found that among 2,204 Chinese college students, 32.71% have hoarding behaviors (Zheng and Liu, 2020). The digital asset that college students hoard the most is photos, and the one they are least willing to delete is also photos. Photos are the main factor causing digital chaos (Broz, 2024). Hoarding digital photos not only causes digital chaos, but may also affect individual work efficiency, bring pressure and anxiety to hoarders, and even trigger cybersecurity issues (Chao and Li, 2023; Wu and Li, 2021). College students lack information literacy and organizational management skills. Studying the hoarding behavior of digital photos among college students can help them manage digital photos correctly, develop healthy digital habits, and avoid the negative effects of digital photo hoarding.

## The current study

Van Bennekom first proposed the concept of digital hoarding, which he believed referred to the accumulation and chaos of digital files, as well as the difficulty of deleting them (Van Bennekom et al., 2015). Subsequently, many scholars have conducted research on digital hoarding. First, the negative impact of digital hoarding behavior. The behavior of digital hoarding will have a certain impact on computer science, psychology, and organizational science, causing problems in information security, information ethics, intellectual property, and so on (Guo et al., 2020; Zhao, 2020, 2025; Xu and Zhang, 2023). Digital hoarding is limited, and the more content is hoarded, the stronger the sense of loss caused by not hoarding content (Schüll, 2018). Digital hoarding behavior can affect individuals' work efficiency, increase psychological pressure and anxiety, and cause network problems (Sweeten et al., 2018; Zhao, 2022). And it will have a certain impact on an individual's cognition, emotions, and behavior. Second, development of a digital hoarding behavior scale. Neave et al. designed a new digital behaviors questionnaire (DBQ), including digital hoarding questionnaire (DHQ) and digital behaviors in the workplace questionnaire (DBWQ). The questionnaire mainly measures individuals' digital hoarding behavior during work (Jia et al., 2022). Based on the context of localization in China, some scholars developed a digital hoarding behavior scale that is tailored to individual characteristics in China (Kirk and Sellen, 2010; Guo et al., 2020; Wu et al., 2021a). Bozaci and Gökdeniz developed a digital photo hoarding behavior scale for individuals who hoard digital photos. Third, research on the influencing factors of digital hoarding behavior. Different scholars have studied the digital hoarding behavior of different individuals. The articles studied the influencing factors of college students' digital hoarding behavior (Wang et al., 2022; Jia et al., 2022; Zhang and He, 2023; Chao and Li, 2023; Guo, 2023; Oravec, 2018). The articles studied the influencing factors of digital hoarding behavior among social media users (Liu and Jia, 2023; Zhang and Liu, 2024a; Zhu and Jiang, 2024).

Digital photos are a type of digital content. Although there have been some studies on digital hoarding behavior at home and abroad, the granularity of research from the perspective of digital hoarding content is relatively coarse, and there is no distinction between digital hoarding content. Digital photos have the maximum share of hoarded digital content (Bozaci and Gökdeniz, 2020). With the increase in the capacity of data storage devices and the reduction in costs, as well as the upgrading of digital photo shooting tools, college students tend to use digital photos to record the little things in their lives. This article specifically studies the factors of digital photo hoarding among college students, as well as the mediating effects of emotional attachment and fear of missing on the relationship between various factors and digital photo hoarding behavior, which is of great significance for healthy digital content management of college students.

## Research model and hypotheses

### Research model

The SOR theory is the Stimulus-Organism-Response (S-O-R) model put forward by Mehrabian and Russell (1974). They believe that behavior is a response made by an individual's psychology influenced by external stimuli and then by the influence of psychology. In this model, the stimulus (S) represents the physical or non-physical stimuli that an individual receives, including those from the external environment, technological progress, and so on. The organism (O) represents the internal states such as cognition and emotion that an individual generates in response to the stimuli. The response (R) represents the behaviors that an individual exhibits after being stimulated. The behavior of digital photo hoarding is a reaction made by an individual due to factors such as the external environment, and it also involves changes in an individual's emotions and cognition. The SOR theory can well construct the relationships among external stimuli, an individual's internal states and behaviors, so it is applicable to the research on the behavior of digital photo hoarding.

**Figure 1:**
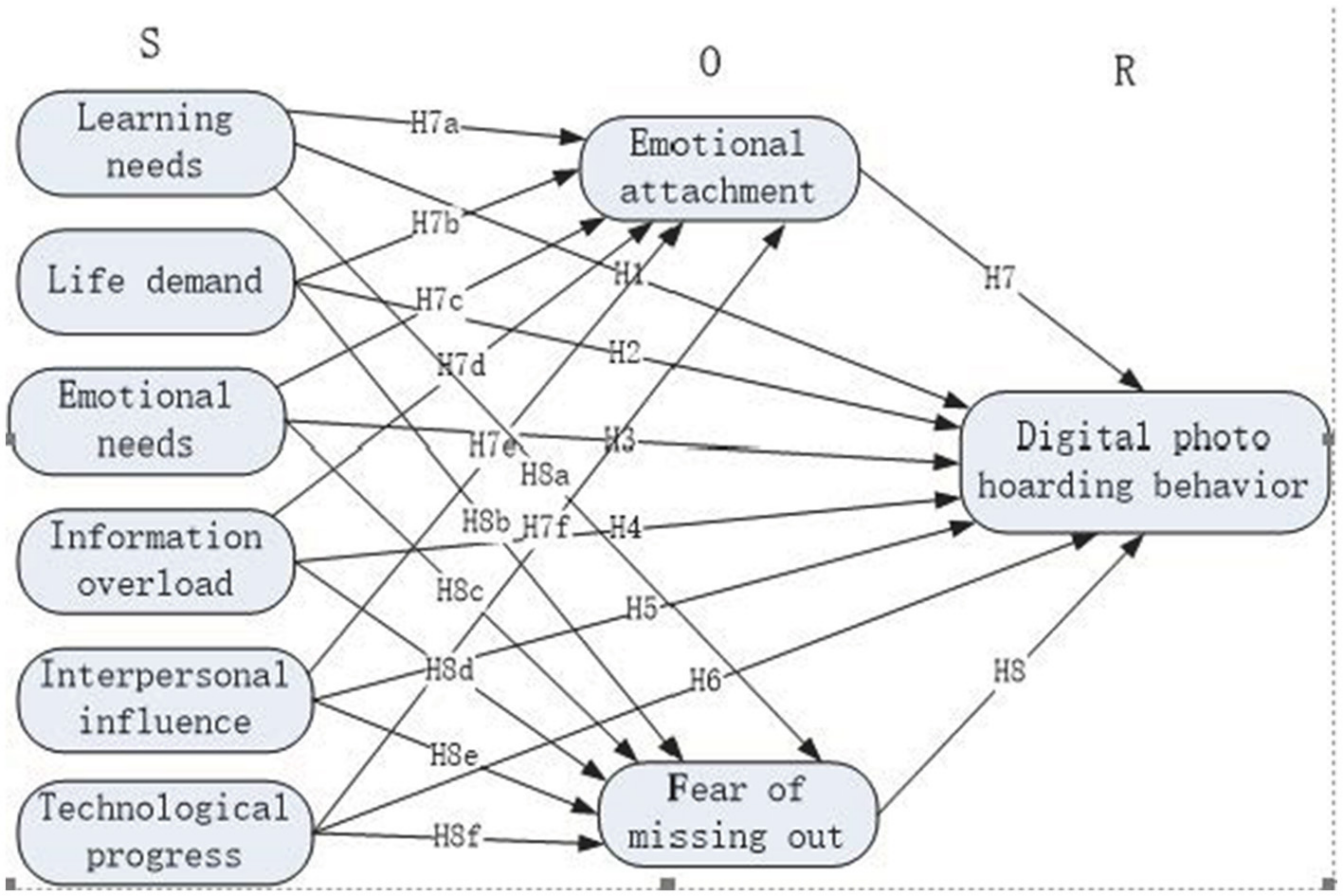
Theoretical model of the influencing factors for college students' digital photo hoarding behavior.

The S-O-R model constitutes a causal chain of external stimuli, user cognition and behavior, and provides a detailed interpretation of the predictive effect of external stimuli on users' emotional responses and subsequent behaviors (Xu et al., 2025). Based on the SOR theory, a model of the influencing factors for college students' digital photo hoarding behavior is constructed, as shown in Figure 1. Among them, learning needs (LN), life demand (LD), emotional needs (EN), information overload (IO), interpersonal influence (II), and technological progress (TP) are regarded as the stimulus (S), emotional attachment (EA) and the fear of missing out (FoMO) are taken as the organism (O), and the digital photo hoarding behavior (DPHB) is considered as the response (R). This theoretical model mainly includes the following two causal relationships: (1) The internal or external stimuli (S) that college students receive directly affect their behavior of hoarding digital photos; (2) The stimuli (S) received by college students have an impact on their digital photo hoarding behavior through the mediating role of organic (O) emotional attachment and fear of missing out.

### Hypotheses formation

#### Learning needs

The learning needs refer to the behavior of college students accumulating photos in order to increase their knowledge reserves or to cope with college assignments, exams, and other such activities. Digital picture hoarding enables people to save comprehensive and well-organized collections of images for various uses, including documentation, study, narrative, and private preservation (Liu et al., 2024). Users' personal needs can make them emotionally attached to data, which in turn affects their behavior. Hoarding data is mainly for academic research, seeking inspiration, and acquiring knowledge (Lu, 2023). Academic demands are an important factor for college students to develop digital hoarding behavior (Lu, 2024; Dai et al., 2024; Liu and Jia, 2023; He and Lin, 2025). Therefore, we proposed the following hypothesis:

H1: Learning needs has a significant positive effect on digital photo hoarding behavior.

#### Life demand

Besides hoarding digital photos due to learning needs, college students may also accumulate digital photos for security and livelihood guarantees. Sweeten et al. believes that future use as evidence is one of the motivations for digital hoarding behavior (Sweeten et al., 2018; Alquista and Baumeister, 2018). For example, they habitually back up data for fear of losing files, take screenshots of shopping and courier information and save them for easy checking at any time, and take pictures of personal identification documents and store them for reference when needed. Anaza and Nowlin believe that individuals tend to hoard important information in order to maintain their own advantages and enhance their competitiveness (Anaza and Nowlin, 2017). Therefore, we proposed the following hypothesis:

H2: Life demand has a significant positive effect on digital photo hoarding behavior.

#### Emotional needs

Individual hoarding behavior is associated with seven beliefs: remembering the past, defining the self, preventing forgetting, fulfilling responsibilities, building a family, maintaining connections with the past, and respecting those who care about us (Luxon et al., 2019). Grisham et al. believes that separation anxiety, uncertainty, interpersonal relationships, and perceived needs can affect data hoarding behavior (Grisham et al., 2019). Some college students hoard digital photos to satisfy their emotional needs. Viewing images of pleasant events, celebrations, and happy times can arouse positive feelings like joy, satisfaction, and appreciation, adding to a feeling of general wellbeing. Individuals hoard digital photos for nostalgia (Zheng and Liu, 2020; Feng, 2022; Fu et al., 2015). Digital photo hoarding can offer a therapeutic avenue for self-reflection, emotional expression, and mental health. Looking through one's digital photo collection can be a soothing and calming hobby. Butcher believes that individuals at work hoard data to gain a sense of security (Butcher, 1995). Therefore, we proposed the following hypothesis:

H3: Emotional needs have a significant positive effect on digital photo hoarding behavior.

#### Information overload

In the era of big data, an overwhelming amount of information is flooding in. Faced with the vast and diverse array of information, college students may accumulate a large number of digital photos due to their inability to organize and process the information they encounter, and they may also choose to store all information out of fear of missing out on important details. When there is too much information, it is difficult to judge the true value of the information. People often increase the frequency of using social media for fear of missing important information (Guo and Peng, 2025; Sun, 2023). There exists a positive association between information overload and the DHB exhibited by college students (Neave et al., 2019). Therefore, we proposed the following hypothesis:

H4: Information overload has a significant positive effect on digital photo hoarding behavior.

#### Interpersonal influence

Interpersonal influence refers to the impact that the environment and people around college students have on them. College students will share interesting images or videos they see on social media with classmates, relatives, and friends. College students enjoy the satisfaction that comes from social interaction when they share the digital photos they have stored. Digital photo hoarding is a helpful tool for improving relationships and is far more than just a habit of collecting photos. Users' social relationships and traditional cultural concepts, among others, can all have an impact on their emotions and behaviors (Lu, 2023). The act of gathering digital images can be a gratifying and relationship-enhancing activity (Agarwal et al., 2024). Additionally, research has found that upward social comparison has a positive impact on digital hoarding behavior (Wang et al., 2023; Liu and Jia, 2023). Therefore, we proposed the following hypothesis:

H5: Interpersonal influence has a significant positive effect on digital photo hoarding behavior.

#### Technological progress

Technological advancements have provided both software and hardware support for college students' digital photo hoarding. The upgrading of photography equipment and the continuous expansion of storage capacities for photos have led college students to not easily delete their favorite photos, and also encourage them to store a large number of digital photos to avoid missing important information (Agarwal et al., 2024). Technical support is one of the fundamental factors that enhance users' attachment to an App (Jin and Hou, 2022). External storage devices, application platforms, and network environments can all have an impact on users' emotions and behaviors (Lu, 2023). Therefore, we proposed the following hypothesis:

H6: Technological progress has a significant positive effect on digital photo hoarding behavior.

#### Mediating effect of emotional attachment

Emotional attachment has a significant impact on digital hoarding behavior (Luxon et al., 2019; Zhang and Liu, 2024b; Wu et al., 2021b). College students may develop emotional attachments to certain things due to their studies, life, and emotional experiences, and rely on technological support to engage in digital photo hoarding behavior. The emotional and personal meaning people attach to their digital photo collections is at the heart of the sentimental value of digital photo hoarding (Agarwal et al., 2024). Emotional attachment plays a mediating role in the impact of personal needs, personal habits, data characteristics, social influence, technical support, and data literacy on data hoarding behavior (Lu, 2023). Therefore, we proposed the following hypothesis:

H7: Emotional attachment mediates the association between (a) learning needs, (b) life demand, (c) emotional needs, (d) information overload, (e) interpersonal influence, (f) technological progress and digital photo hoarding behavior.

#### Mediating effect of fear of missing out

The fear of missing out (FoMO) is an important internal factor leading to digital photo hoarding among college students. College students may engage in digital photo hoarding behavior due to their academic, lifestyle, and emotional needs, while technological support, interpersonal influence, and information overload can exacerbate this behavior. The fear of missing out mediates the impact of upward social comparison on digital hoarding behavior (Wang et al., 2023; Liu and Jia, 2023). Therefore, we proposed the following hypothesis:

H8: Fear of missing out mediates the association between (a) learning needs, (b) life demand, (c) emotional needs, (d) information overload, (e) interpersonal influence, (f) technological progress and digital photo hoarding behavior.

## Research method

### Survey development and data collection

Referencing existing digital hoarding scales both domestically and internationally, and combining semi-structured interviews, the items for this questionnaire survey were ultimately determined after a preliminary research. The questionnaire uses a Likert five-point scale (ranging from 1 to 5, representing “strongly disagree,” “disagree,” “neutral,” “agree,” and “strongly agree”) to measure the respondents' level of agreement with the items.

The questionnaire is divided into two parts. The first part mainly collects basic information about the respondents, including gender, grade, etc. The second part mainly investigates the factors influencing digital photo hoarding, including 9 observed variables: learning needs (LN), life demand (LD), emotional needs (EN), information overload (IO), interpersonal influence (II), technological progress (TP), emotional attachment (EA), fear of missing out (FoMO), and digital photo hoarding behavior (DPHB). The items for each observed variable and their reference sources are shown in Appendix 1.

Data collection was primarily conducted online, using QuestionStar to create the finalized questionnaire, which was then distributed to college students. After excluding invalid questionnaires, a total of 294 questionnaires were collected, with a response rate of 98%.

## Results

### Measurement model

The assessment of the measurement model encompassed an evaluation of its reliability, convergent validity, and discriminant validity. The reliability and validity of the measurement model were examined using the SmartPLS 4.0, as follows:

#### Reliability

Conducting reliability testing on the questionnaire can reveal the level of consistency. The measurement indicators include Cronbach's Alpha coefficient and Composite Reliability (CR). When Cronbach's Alpha is between 0.7 and 0.8, it indicates that the overall reliability of the questionnaire meets the requirements. When Cronbach's Alpha is >0.8, it indicates that the overall reliability of the questionnaire is good (Zaremohzzabieh et al., 2024). When Composite Reliability is >0.7, it indicates that the composite reliability of the questionnaire is good. Table 1 presents the Cronbach's Alpha, Composite Reliability, and Average Variance Extracted (AVE) for the questionnaire. From Table 1, it can be seen that the Cronbach's Alpha for all variables is >0.7, with 7 out of 8 variables having a Cronbach's Alpha coefficient above 0.8, and the Composite Reliability is >0.8 for all variables, indicating that the questionnaire has good reliability.

**Table 1:** The reliability of the measurement.

**Constructs**	**Items**	**Factor loading**	**Cronbach's alpha**	**CR**	**AVE**	**VIF**
DPHB	DPHB1	0.765	0.895	0.920	0.657	1.931
	DPHB2	0.789				2.026
	DPHB3	0.852				2.735
	DPHB4	0.852				3.158
	DPHB5	0.849				3.401
	DPHB6	0.749				1.905
EA	EA1	0.880	0.920	0.944	0.807	2.786
	EA2	0.919				3.791
	EA3	0.881				2.746
	EA4	0.912				3.454
EN	EN1	0.910	0.944	0.957	0.818	4.809
	EN2	0.898				4.255
	EN3	0.916				4.630
	EN4	0.894				3.752
	EN5	0.904				3.803
FOMO	FoMO1	0.895	0.883	0.919	0.740	2.940
	FoMO2	0.832				1.976
	FoMO3	0.855				2.212
	FoMO4	0.859				2.392
II	II1	0.749	0.753	0.859	0.671	1.344
	II2	0.868				1.903
	II3	0.836				1.677
IO	IO1	0.897	0.896	0.935	0.828	3.077
	IO2	0.890				2.328
	IO3	0.943				4.052
LN	LN1	0.858	0.725	0.878	0.783	1.478
	LN2	0.910				1.478
LD	LD1	0.932	0.839	0.925	0.861	2.091
	LD2	0.923				2.091
TP	TP1	0.827	0.899	0.926	0.714	2.187
	TP2	0.796				2.021
	TP3	0.859				2.617
	TP4	0.890				3.334
	TP5	0.850				2.649

To assess multicollinearity, we also conducted a check on the Variance Inflation Factor (VIF). As shown in Table 1, all VIF values are below the recommended threshold of 5, confirming that there is no multicollinearity in the research model.

#### Validity

Validity assessment can reveal the effectiveness of a questionnaire. The indicators of measurement validity include convergent validity and discriminant validity. Convergent validity is measured by the Average Variance Extracted (AVE). When the AVE is >0.5, it indicates good convergent validity (Zaremohzzabieh et al., 2024). Discriminant validity can be measured using the Fornell-Larcker Criterion and cross-loadings. As shown in Table 2, the values on the diagonal represent the square root of the AVE for each variable; the squared root of the AVE for each construct is greater than its correlation coefficients with other constructs. The item loadings of each construct are significantly higher than the cross-loadings of other constructs (Due to space constraints, the cross-loadings are not attached and can be requested from the authors upon request.). Therefore, the questionnaire has good discriminant validity.

**Table 2:** Square root of construct's AVE and its correlation with any other construct.

**Constructs**	**DPHB**	**EA**	**EN**	**FoMO**	**II**	**IO**	**LN**	**LD**	**TP**
DPHB	**0.811**								
EA	0.749	**0.898**							
EN	0.517	0.740	**0.904**						
FoMO	0.782	0.832	0.664	**0.860**					
II	0.638	0.684	0.693	0.720	**0.819**				
IO	0.537	0.648	0.772	0.666	0.756	**0.910**			
LN	0.434	0.545	0.669	0.580	0.555	0.678	**0.885**		
LD	0.445	0.641	0.838	0.587	0.645	0.785	0.712	**0.928**	
TP	0.622	0.697	0.731	0.716	0.737	0.797	0.660	0.737	**0.845**

Bold values indicates the AVE >0.5.

#### Structural model

With the Bootstrapping in SmartPLS 4.0, the hypothesis testing results shown in Tables 3–5 were obtained.

**Table 3:** Total effect.

**Path**	**Coefficients**	***T-values***	***P-values***	**Result**
EA -> DPHB	0.392^***^	4.567	0.000	Significant
EN -> DPHB	0.137	1.428	0.153	Insignificant
EN -> EA	0.484^***^	5.397	0.000	Significant
EN -> FoMO	0.243	2.757	0.006	Significant
FoMO-> DPHB	0.440^***^	5.146	0.000	Significant
II -> DPHB	0.430^***^	4.209	0.000	Significant
II -> EA	0.278^**^	3.402	0.001	Significant
II -> FoMO	0.381^***^	5.296	0.000	Significant
IO -> DPHB	−0.030	0.282	0.778	Insignificant
IO -> EA	−0.059	0.663	0.508	Insignificant
IO -> FoMO	0.036	0.388	0.698	Insignificant
LD -> DPHB	−0.182^*^	2.007	0.045	Significant
LD -> EA	−0.039	0.439	0.661	Insignificant
LD -> FoMO	−0.155	1.822	0.068	Insignificant
LN -> DPHB	0.063	1.033	0.302	Insignificant
LN -> EA	0.022	0.392	0.695	Insignificant
LN -> FoMO	0.152	1.851	0.064	Insignificant
TP -> DPHB	0.300^***^	3.622	0.000	Significant
TP -> EA	0.177^*^	2.116	0.034	Significant
TP -> FoMO	0.220^**^	2.744	0.006	Significant

^*^indicates *p* < 0.05; ^**^indicates *p* < 0.01; ^***^indicates *p* < 0.001.

#### Total effect

The total effect measures the entire influence of one variable on another, including the direct effect (represented by the path coefficient) and the indirect effect. It can comprehensively assess the importance of one variable to another, especially in complex models where there are mediator variables. It helps to understand how one variable influences another through multiple pathways. As shown in Table 3, emotional attachment, fear of missing out, interpersonal influence, life demand, and technological progress are important influencing factors for college students' digital photo hoarding behavior.

#### Path coefficients and specific indirect effects

The path coefficient represents the strength of the direct causal relationship between variables. In a path model, the arrow from an independent variable (predictor variable) to a dependent variable (predicted variable) represents a hypothesized causal connection, and the path coefficient is the quantification of the strength of this connection. As shown in Table 4, the results show that emotional attachment has a significant positive effect on digital photo hoarding. Emotional need, interpersonal influence, and technological progress have significant positive effects on emotional attachment. Hence, H7, H7c, H7e, H7f are supported. Fear of missing out has a significant positive effect on digital photo hoarding. Emotional need, interpersonal influence, and technological progress have significant positive effect on fear of missing out. Hence, H8, H8c, H8e, H8f are supported.

**Table 4:** Parameter estimates for the path model predicting digital photo hoarding behavior.

**Hypothesis**	**Path**	**Coefficients**	***T-values***	***P-values***	**Supported**
H1	LN -> DPHB	−0.013	0.198	0.843	No
H2	LD -> DPHB	−0.098	1.281	0.200	No
H3	EN -> DPHB	−0.159	2.343	0.019	No
H4	IO -> DPHB	−0.023	0.256	0.798	No
H5	II -> DPHB	0.154	1.760	0.078	No
H6	TP -> DPHB	0.133	1.721	0.085	No
H7	EA -> DPHB	0.392^***^	4.567	0.000	Yes
H7a	LN -> EA	0.022	0.392	0.695	No
H7b	LD -> EA	−0.039	0.439	0.661	No
H7c	EN -> EA	0.484^***^	5.397	0.000	Yes
H7d	IO -> EA	−0.059	0.663	0.508	No
H7e	II -> EA	0.278^**^	3.402	0.001	Yes
H7f	TP -> EA	0.177^*^	2.116	0.034	Yes
H8	FoMO-> DPHB	0.440^***^	5.146	0.000	Yes
H8a	LN -> FoMO	0.152	1.851	0.064	No
H8b	LD -> FoMO	−0.155	1.822	0.068	No
H8c	EN -> FoMO	0.243^**^	2.757	0.006	Yes
H8d	IO -> FoMO	0.036	0.388	0.698	No
H8e	II -> FoMO	0.381^***^	5.296	0.000	Yes
H8f	TP -> FoMO	0.220^**^	2.744	0.006	Yes

^*^indicates *p* < 0.05; ^**^indicates *p* < 0.01; ^***^indicates *p* < 0.001.

**Figure 2:**
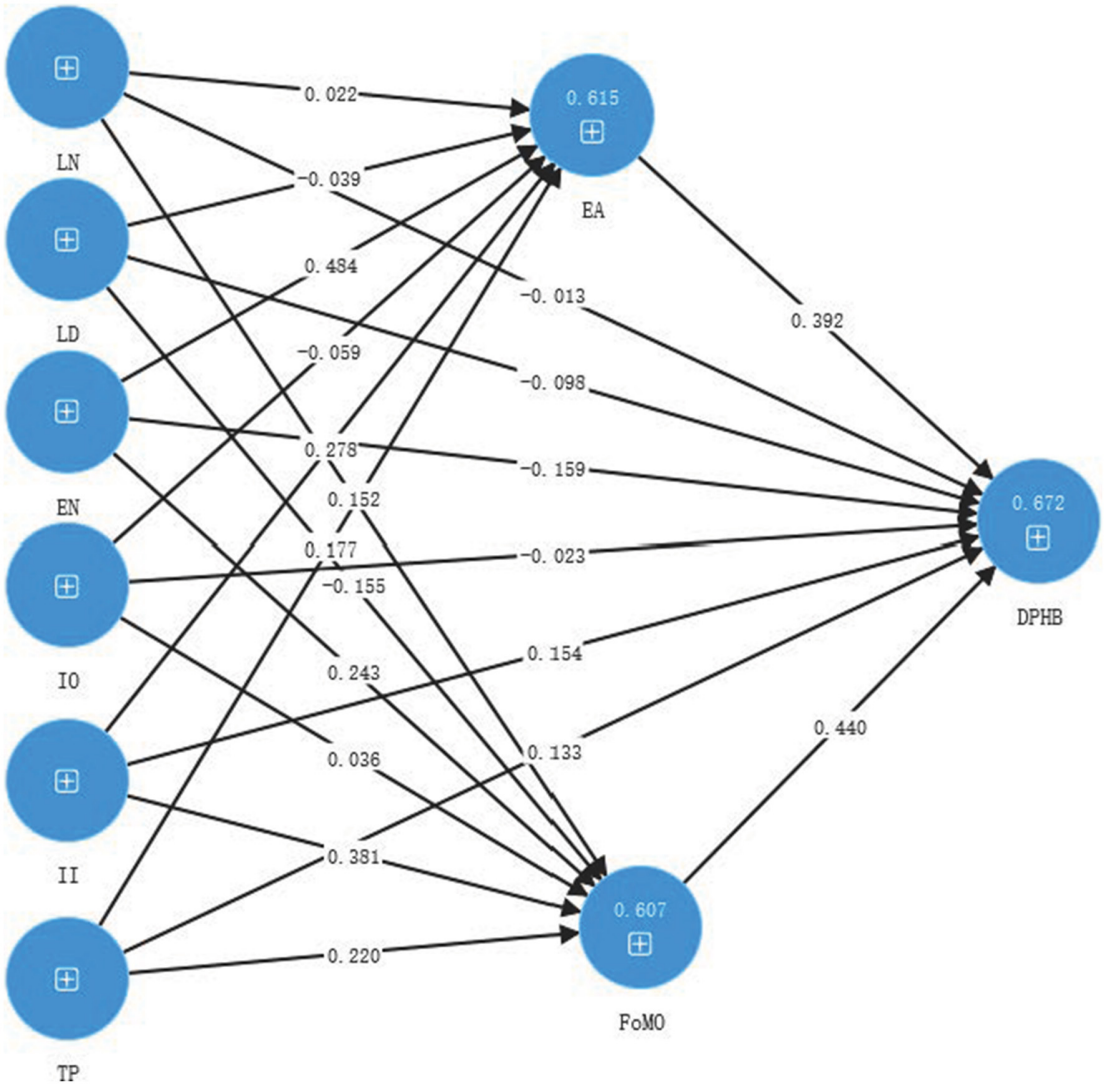
Results from the PLS model.

As shown in Table 5 and Figure 2, both emotional attachment and fear of missing out mediate the relationship between emotional need, interpersonal influence, and technological progress with digital photo hoarding behavior.

**Table 5:** The mediation effects on digital photo hoarding behavior.

**Path**	**Coefficients**	***T-values***	***P-values***	**Supported**
EN -> EA -> DPHB	0.190^**^	3.260	0.001	Yes
II -> FoMO -> DPHB	0.167^**^	3.439	0.001	Yes
IO -> FoMO -> DPHB	0.016	0.370	0.712	No
II -> EA -> DPHB	0.109	2.789	0.005	Yes
LD -> FoMO -> DPHB	−0.068	1.779	0.075	No
IO -> EA -> DPHB	−0.023	0.647	0.518	No
LN -> FoMO -> DPHB	0.067	1.847	0.065	No
LD -> EA -> DPHB	−0.015	0.431	0.667	No
TP -> FoMO -> DPHB	0.097^*^	2.382	0.017	Yes
LN -> EA -> DPHB	0.009	0.394	0.694	No
TP -> EA -> DPHB	0.069^*^	1.893	0.048	Yes
EN -> FoMO -> DPHB	0.107^*^	2.539	0.011	Yes

^*^indicates *p* < 0.05; ^**^indicates *p* < 0.01; ^***^indicates *p* < 0.001.

Figure 2 is a theoretical model with path coefficients based on the structural equation model.

## Discussion

The article examines the influencing factors affecting digital photo hoarding among college students and the mediating roles of emotional attachment and fear of missing out in it. Through a questionnaire survey and using structural equation modeling, it is found that interpersonal influence, life demand, technological progress, emotional attachment, and fear of missing out have a significant impact on digital photo hoarding behavior. Learning needs, emotional needs, information overload have no significant impact on the hoarding behavior of digital photos. Moreover, both emotional attachment and fear of missing out mediate the effects of emotional need, interpersonal influence, and technological progress on digital photo hoarding behavior.

College students are surrounded by classmates, relatives, and friends who influence their digital photo hoarding behavior. College students choose to hoard digital photos because of peer comparisons or in order to keep good memories with family and friends, which is consistent with previous research that upward comparisons have a significant effect on digital hoarding behavior (Liu and Jia, 2023) and that people choose not to delete photos in order to keep the good moments (Agarwal et al., 2024). Life demand also lead to digital photo hoarding, and college students will choose not to delete digital photos for a long time in order to keep evidence, credentials, etc. (Liu and Jia, 2023). Technological progress provides convenient conditions for digital photo hoarding, and the expansion of storage space, shrinking costs, and the convenience of cross-platform storage all provide conditions for college students to hoard digital photos, which is consistent with the results that perceived low price and perceived convenience have a positive effect on digital hoarding behavior (Vinoi et al., 2024). Learning needs, emotional needs and information overload have no direct impact on college students' behavior of hoarding digital photos. It is indicated that the hoarding behavior of digital photos among college students will not be affected by learning needs and information overload. However, emotional needs influence college students' behavior of hoarding digital photos by affecting emotional attachment and fear of missing out. The emotional and personal meaning people attach to their digital photo collections is at the heart of the sentimental value of digital photo hoarding. Gratification derived from digital photo attachment can strongly induce people to gather and store vast collections of digital photos (Agarwal et al., 2024).

It was also found that emotional attachment and fear of missing out mediate the effects of emotional need, interpersonal influence, and technological progress on digital photo hoarding behaviors. Many scholars in the past have used emotional attachment and fear of missing out as mediators in the study of digital hoarding behaviors. For example, emotional attachment mediates the effects of personal habit, personal need, social influence, and technological support on digital hoarding behaviors (Lu, 2023). Fear of missing out mediates the effect of upward social comparison on digital hoarding behavior (Liu and Jia, 2023; Wang et al., 2023).

## Implication

### Theoretical implication

Although scholars at home and abroad have conducted research on topics related to digital hoarding, most of them have studied the influencing factors of digital hoarding behavior and the moderating and mediating effects of different factors on digital hoarding behavior. Fewer studies have been conducted on specific digital hoarding content. In the new media era, with the upgrading of storage devices, storage space, and photographic technology, digital photographs are the type of data with more digital content storage. Studying the factors influencing college students' digital photo hoarding behaviors and the mediating roles of emotional attachment and misplaced fear in them has several theoretical implications:

First, it provides a new research perspective for digital hoarding research, different groups hoard different types of digital content, and this study provides reference and reflection for refining digital hoarding content research.

Second, based on the SOR model, structural equation modeling is used to study the influencing factors of digital photo hoarding behavior and the mediating role of emotional attachment and fear of missing out, which provides theoretical and methodological reference for digital hoarding behavior research.

Third, not only study the influencing factors of digital hoarding behavior, but also analyze the mediating role played by multimediating variables in the influence of emotional need, interpersonal influence and technological progress on digital photo hoarding behavior.

### Practical implications

This study has not only some theoretical significance, but also some practical significance. College students will engage in digital photo hoarding because of comparisons with classmates around them, as well as because of the demands of life, and will also be too lazy to delete photos simply because of the convenience and low cost of storage. The massive hoarding of digital photos may cause the leakage of information, and may also bring some anxiety and pressure to college students. Therefore, it is necessary to organize digital photos at regular intervals, and college students should store digital photos reasonably, make rational use of digital resources, and develop good digital habits.

## Limitations and directions for future research

Although this study has certain contributions, there are some shortcomings. First of all, the study's research subjects are college students, which is not generalizable. Future studies should include research subjects from different backgrounds and cultures, either individuals or organizations. For example, the hoarding behavior of digital photos by postgraduate students, research institutions, data resource management departments, etc. Study the differences in digital hoarding behaviors among college students in different countries. Secondly, the study only analyzed the behavior of mediating variables on digital photo hoarding behavior, and did not involve the study of moderating variables, which can be studied in the future to investigate the influence of moderating factors on digital photo hoarding behavior. For example, the moderating effect of conservative on the association between emotional attachment and digital hoarding behavior. Thirdly, there are certain errors in the data collected through questionnaires. For instance, the respondents deliberately choose the “socially expected answers” to conform to social norms, gain recognition from others, or avoid negative evaluations, rather than their true thoughts or behavioral tendencies. Or, due to factors such as self-awareness, motivation, or context, there may be deviations where the reported content does not match the actual behavior. Future research can combine objective data verification to improve the accuracy of source data. Fourthly, with the change of time and technology, the factors affecting the digital photo hoarding behavior of college students may change, and future research can explore other factors affecting the digital photo hoarding behavior on the basis of this study, and also study the relationship between digital photo hoarding behavior and physical hoarding behavior.

## Conclusion

This article takes college students as the research object and studies their hoarding behavior of digital photos based on the SOR model. By using the structural equation model and conducting a survey among 294 college students, it was found that interpersonal influence, life demand, and technological progress have an important impact on college students' digital photo hoarding. Emotional attachment mediates the relationship between emotional needs, interpersonal influence, and technological progress with digital photo hoarding behaviors. Fear of missing out mediates the association between emotional needs, interpersonal influence, and technological progress, and digital photo hoarding behavior. Hence, H7, H7c, H7e, H7f, H8, H8c, H8e, H8f are supported. Although the research has certain limitations, it also makes certain theoretical and practical contributions, which not only broaden the research direction for the study of digital hoarding, but also help to guide college students to use digital content correctly, improve digital literacy, and develop good digital governance habits.

## Data Availability

The original contributions presented in the study are included in the article/Supplementary material, further inquiries can be directed to the corresponding author.
