# Supplementary material for: Exploration of factors of digital photo hoarding behavior among university students and the mediating role of emotional attachment and fear of missing out
Source: Front Psychol. 2025 Sep 15;16:1607274. doi: 10.3389/fpsyg.2025.1607274 (PMC12477202; doi:10.3389/fpsyg.2025.1607274)
Supplement: Supplementary file 1 [file Supplementary_file_1.docx]

Appendix Measures of variables

| Constructs | Items | Sources |
| --- | --- | --- |
| learning needs (LN) | LN1: Hoarding photos facilitates browsing and accessing them during my studies. | Wu（2021）、Lu2023） |
|  | LN2: Hoarded photos allow me to extract information and knowledge to complete assignments and exams. |  |
|  | LN3: I take photos and save all content that I believe will be useful in the future. |  |
| emotional needs (EN) | EN1: Viewing old photos relaxes and comforts me. | Bozacı（2020）、Wu（2021） |
|  | EN2: Looking at past photos can reduce my stress. |  |
|  | EN3: When I encounter something interesting, I habitually take a photo of it. |  |
|  | EN4: Photos can share happiness. |  |
|  | EN5: Taking new photos makes me very happy. |  |
| information overload(IO) | IO1: There is so much information online that I don't have time to read it carefully, so when I see useful information, I save it by taking a screenshot. | Zeinab et al. （2023）、Sun（2023） |
|  | IO2: When a platform recommends content that interests me, I save it by taking a screenshot. |  |
|  | IO3: Information online updates too quickly, and for some hard-to-obtain, time-sensitive content, I save it by taking a photo. |  |
| interpersonal influence(II) | II1: When my classmates take photos, I also take photos, regardless of their usefulness. | Lu（2023）、Wang（2023）、Liu（2023）、Agarwal（2024） |
|  | II2: I save useful (interesting) photos shared by my classmates. |  |
|  | II3: When I encounter something that my classmates would be interested in, I take a photo and send it to them. |  |
| technological progress(TP) | TP1: With technological advancements, the cost of photo storage is low. | Lu（2023）、Sun（2023）、Agarwal et al.（2024） |
|  | TP2: If there is enough storage space, I generally do not delete the photos I save. |  |
|  | TP3: Photos can be taken, accessed, and retrieved at any time. |  |
|  | TP4: Saving photos is simple, and I can upload, forward, share, and transfer photos in batches. |  |
|  | TP5: I can save pictures across platforms and software. |  |
| emotional attachment(EA) | EA1: My emotional attachment to photos is like my emotional attachment to certain people. | Neave et al.（2019）、Lu（2023） |
|  | EA2: Photos are precious memories, and I am reluctant to delete them. |  |
|  | EA3: If a photo is accidentally deleted, I would be very upset. |  |
|  | EA4: Photos provide me with emotional comfort. |  |
| fear of missing out (FoMO) | FoMO1: If I don't save important photos, I feel like I'm missing out on important content. | Neave et al.（2019）、Lu（2023） |
|  | FoMO2: To not miss out on important study content, I take photos of everything and save them. |  |
|  | FoMO3: I'm afraid I won't be able to find certain content later, so I take photos or screenshots to save it. |  |
|  | FoMO4: If I don't save an important photo, I feel anxious. |  |
| digital photo hoarding behavior(DPHB) | DPHB1: I may not look at many photos, but I also don't want to delete them. | Neave et al.（2019）、Jia（2022） |
|  | DPHB2: Sometimes when I want to find a certain photo, it's hard to find because I have too many photos. |  |
|  | DPHB3: Deleting photos stresses me out. |  |
|  | DPHB4: Deleting certain photos feels like losing a part of me. |  |
|  | DPHB5: When deleting photos, I'm very indecisive about which ones to delete. |  |
|  | DPHB6: Deleting photos takes up a lot of my time. |  |
